# Cardiovascular Disease, Cancer and Dementia in Diabetes: 20‐Year Trends Will Necessitate New Treatment Priorities

**DOI:** 10.1111/1753-0407.70230

**Published:** 2026-05-04

**Authors:** Zachary T. Bloomgarden

Cardiovascular disease (CVD) has been considered the dominant cause of mortality in people with diabetes. The 2013 European Society of Cardiology guidelines cited estimates that approximately 70% of individuals with diabetes die from CVD [1], although a number of studies have demonstrated substantial declines in diabetes‐related complication rates [2] and proportional mortality rates [3].

An important study by Magliano and colleagues examines whether these trends are consistent across multiple high‐income settings, and quantifies the parallel trajectories of CVD, cancer and dementia mortality in people with versus without diabetes, with analysis of 1.7 billion person‐years of observation with approximately 2.7 million deaths in people with diabetes and 11 million in those without, allowing us updated perspective on the degree of CVD dominance in diabetes mortality over 23 year observation windows [1]. The findings have direct implications for clinical practice, public health planning, and research prioritization. The analysis spans study periods from 2000 through 2023 in Finland, with complementary data from 10 other countries, capturing a period of CVD decline, with increasing use of cardioprotective pharmacotherapy including statins, antihypertensives, SGLT2 inhibitors, and GLP‐1 receptor agonists, along with information about multiple additional causes of mortality, particularly cancer and dementia, based on whole‐population administrative and registry datasets not susceptible to the referral, survival, and ascertainment biases that affect clinical trial populations and voluntary registries, with complete national vital statistics registration in these jurisdictions monitored. Using age‐sex standardized rates restricted age 40–89 guards against the confounding of birth‐cohort effects with period trends. By studying absolute mortality rates along with mortality rate ratios (MRRs) comparing people with and without diabetes, the authors disentangle absolute progress from changes in relative excess risk. Using the large language model Claude Sonnet 4.6 [4], we provide an independent quantitative perspective based on mortality rates extracted from the paper's published log‐scale figures and from supplementary table 6 of the Magliano et al. appendix [1], weighted by country person‐years to produce cross‐jurisdictional summary estimates. These values should be understood as approximations (with inherent imprecision from figure‐reading) rather than replacements for the paper's modeled age‐period‐cohort analysis estimates; they are directionally informative and serve to contextualize the paper's findings (see Appendix S1 for prompts used and full analyses).

## 1 Concordance With the Paper's Findings

Tables 1, 2, 3 show declines in weighted‐mean CVD, cancer and dementia mortality declines both among people with diabetes and those not having diabetes, particularly dramatic for CVD, amounting to 69% and 72% respectively over the two‐decade observation window. This is fully consistent with the paper's primary finding of CVD mortality declines in both groups [1]. Table 4 shows a CVD mortality rate ratio (MRR) that is stable to slightly rising (1.77 in 2000 to 1.97 in 2020), corroborating the paper's conclusion that excess relative CVD risk in diabetes has not materially improved [1]. Table 5 quantitates a finding that the paper does not explicitly present: the CVD/cancer mortality rate ratio within the diabetes population fell from 2.56 in 2000 to exactly 1.00 in 2020, while in the non‐diabetes population it fell to 0.71—meaning cancer deaths already exceed CVD deaths among people without diabetes in these high‐income settings. The dementia MRR in Table 4 (weighted means ranging from 1.04 to 1.28) is notably lower than the CVD MRR (~1.8–2.0) and cancer MRR (~1.4–1.6), perhaps attributable to competing cause effects, in which excess competing mortality suppresses apparent mortality rates from competing causes. The paper does not directly report CVD/cancer, CVD/dementia, or cancer/dementia rate ratios over time. Table 6 shows the CVD/dementia ratio in the diabetes population falling from approximately 11 in 2000 to approximately 4–7 by 2020, driven by the simultaneous marked CVD decline and the gradual but consistent rise in dementia.

**TABLE 1:** Cancer mortality per 1000 person‐years.

Year	With diabetes (wt. mean)	Without diabetes (wt. mean)
2000	9.0	6.5
2005	9.5	6.2
2010	8.6	5.6
2015	7.5	5.2
2020	7.1	5.2

**TABLE 2:** CVD mortality per 1000 person‐years.

Year	With diabetes (wt. mean)	Without diabetes (wt. mean)
2000	23.0	13.0
2005	14.3	7.8
2010	11.1	5.9
2015	8.7	4.5
2020	7.2	3.7

**TABLE 3:** Dementia mortality per 1000 person‐years.

Year	With diabetes (wt. mean)	Without diabetes (wt. mean)
2000	2.1	2.0
2005	1.3	1.2
2010	1.7	1.3
2015	1.5	1.3
2020	1.7	1.4

**TABLE 4:** Mortality rate ratios (DM vs. No‐DM), weighted means.

Year	Cancer MRR	CVD MRR	Dementia MRR
2000	1.38	1.77	1.05
2005	1.55	1.80	1.04
2010	1.57	1.85	1.28
2015	1.47	1.98	1.16
2020	1.40	1.97	1.05

**TABLE 5:** CVD/cancer mortality rate ratio over time.

Year	With diabetes	Without diabetes
2000	2.56	2.00
2005	1.54	1.24
2010	1.30	1.06
2015	1.16	0.86
2020	1.00	0.71

**TABLE 6:** CVD/dementia and cancer/dementia ratios (with diabetes, weighted means).

Year	CVD/dementia ratio	Cancer/dementia ratio
2000	10.95	4.29
2005	12.29	8.98
2010	6.53	5.06
2015	5.80	5.00
2020	4.24	4.18

## 2 Clinical and Public Health Implications

### 2.1 Cardiovascular Disease

The ~69% decline in weighted‐mean CVD mortality in people with diabetes over approximately 20 years represents one of the most impressive achievements in the management of any chronic non‐communicable disease in recorded epidemiology. The compounding effects of population‐level smoking cessation, widespread statin and antihypertensive therapy, improved glycemic management, and most recently the introduction of SGLT2 inhibitors and GLP‐1 receptor agonists account collectively for this trajectory. However, the absolute CVD mortality rate in people with diabetes (~7.2/1000 PY in 2020) remains approximately double that of people without diabetes (~3.7/1000 PY), and the MRR of approximately 1.8–2.0 has not narrowed despite two decades of therapeutic progress. We should bear in mind that CVD mortality is increasing as a contributing cause of death in younger age groups [5], a finding that is not captured by the 40–89 year age restriction of the current analysis and which represents a potentially emerging countertrend. Younger‐onset patients with type 2 as well as with type 1 diabetes are likely to have substantially different risk profiles for cancer, dementia, and CVD, and future analyses separating the two types and specifically following people with younger onset Type 2 diabetes will be important. Furthermore, the overall decline in CVD mortality across both diabetes and non‐diabetes groups means that future improvements will be harder to achieve. The study covered 11 high‐income, predominantly English‐speaking or Western European populations, with the addition of South Korea. Other East Asian, South Asian, Latin American, sub‐Saharan African, and Middle Eastern populations collectively account for the majority of the global diabetes burden and are characterized by younger age at onset, greater degrees of hyperglycemia, lower rates of preventive pharmacotherapy access, and different cancer and infectious disease epidemiology, and it will be crucial to replicate the study in these other populations, as well as ascertaining more individual‐level information about obesity, glycemic control, smoking, lipid levels, statin and antihypertensive and novel pharmacotherapy utilization, and socioeconomic distribution.

### 2.2 Cancer

Cancer had become the leading cause of death in people with diabetes in four of the 11 high‐income jurisdictions, with declines in only 3 of 9 diabetes populations examined, compared with 6 of 9 non‐diabetes populations. The persistent cancer MRR of ~1.4–1.6 reflects effects of hyperinsulinemia and insulin resistance, low‐grade inflammation, obesity, and reduced access to or uptake of cancer screening programs among individuals with multiple comorbidities. Cancer surveillance and prevention strategies specifically targeting people with diabetes, including adherence to recommended screening intervals for colorectal, breast, cervical, and hepatocellular cancers, need to be embedded in clinical practice guidelines in the same manner as cardiovascular risk management, an asymmetry not tenable given the current proportional mortality data.

### 2.3 Dementia

The finding of rising dementia mortality in 6 of 7 eligible jurisdictions, independent of population aging in the 40–89 year standardized analysis, may be the most clinically challenging aspect of the mortality landscape in diabetes. The low dementia MRR (1.0–1.3) should not be interpreted as evidence that diabetes does not increase dementia risk [6]; the low MRR in the mortality context likely reflects the competing cause dynamics described above, combined with the fact that dementia in people with diabetes is attributable to a combination of diabetes‐specific and diabetes‐independent pathways that are not fully separable at the population level. An important further issue is that dementia may be under‐ascribed as a cause of death, leading to lower apparent mortality rates.

### 2.4 Health Systems and Guidelines

The outdated assertion that approximately 70% of people with diabetes die from CVD—still found in textbooks and cited in clinical materials—should be retired. The proportional mortality data show that CVD was the leading cause in only 64% of jurisdictions at end of observation, down from 82% at baseline, and that at no jurisdiction did CVD account for more than 50% of deaths by the end of the observation window. The shift to a mixed‐cause mortality landscape—CVD, cancer, and dementia all contributing materially—requires corresponding adjustment in guideline priorities, surveillance frameworks, and research funding allocation. The 70% figure was an approximation grounded in evidence from an earlier era, and it is no longer an accurate description of diabetes mortality in high‐income countries.

## 3 Conclusions

The study by Magliano and colleagues [1] provides comprehensive multinational evidence that CVD is no longer universally the leading cause of death in people with diabetes in high‐income settings. This is not to discount the relative CVD excess of people with diabetes, with a MRR of approximately 1.8–2.0 throughout the observation period, with the consequent gap between diabetes and non‐diabetes CVD mortality. However, rising dementia mortality and persistent excess cancer mortality will mark the next frontier in diabetes complications management. The current analysis (Tables 1, 2, 3, 4, 5, 6) using data extracted from the paper's published figures and supplementary tables [1] corroborates all the major findings. The CVD/cancer rate ratio in people with diabetes fell from 2.56 in 2000 to 1.00 in 2020, and in the non‐diabetes population that ratio has already fallen to 0.71, meaning cancer deaths now exceed CVD deaths. We need to recalibrate clinical guidelines, population surveillance priorities, and research investment in diabetes complications.

## Funding

The author has nothing to report.

## Conflicts of Interest

The author declares no conflicts of interest.

## Supporting information


**Appendix S1:** Supporting Information

## References

[1] D. J. Magliano , J. I. Morton , L. Chen , et al., “Trends in Cause‐Specific Mortality Among People With and Without Diabetes in High‐Income Settings: A Multinational, Population‐Based Study,” Lancet Diabetes and Endocrinology 14, no. 5 (2026): 380–389, 10.1016/S2213-8587(25)00398-59. 41819111

[2] E. W. Gregg , Y. J. Cheng , M. Srinivasan , et al., “Trends in Cause‐Specific Mortality Among Adults With and Without Diagnosed Diabetes in the USA: An Epidemiological Analysis of Linked National Survey and Vital Statistics Data,” Lancet 391, no. 10138 (2018): 2430–2440. 29784146 10.1016/S0140-6736(18)30314-3

[3] J. Pearson‐Stuttard , J. Bennett , Y. J. Cheng , et al., “Trends in Predominant Causes of Death in Individuals With and Without Diabetes in England From 2001 to 2018: An Epidemiological Analysis of Linked Primary Care Records,” Lancet Diabetes and Endocrinology 9, no. 3 (2021): 165–173. 33549162 10.1016/S2213-8587(20)30431-9 PMC7886654

[4] Anthropic , “Claude 4.6 Sonnet [Large Language Model],” 2026, https://www.anthropic.com.

[5] Y. H. Yeo , B. J. San , G. K. Lim , et al., “Increasing Cardiovascular Mortality in Young Adults With Diabetes Mellitus as a Contributing Cause in the United States,” Diabetes, Obesity & Metabolism 28, no. 4 (2026): 2672–2681. 10.1111/dom.70441 41491591

[6] S. Liu , “Geng D.A Systematic Analysis for Disease Burden, Risk Factors, and Trend Projection of Alzheimer's Disease and Other Dementias in China and Globally,” PLoS One 20, no. 5 (2025): e0322574. 40333703 10.1371/journal.pone.0322574 PMC12057861

