# Supplementary material for: Cardiovascular Disease, Cancer and Dementia in Diabetes: 20‐Year Trends Will Necessitate New Treatment Priorities
Source: J Diabetes. 2026 May 4;18(5):e70230. doi: 10.1111/1753-0407.70230 (PMC13138851; doi:10.1111/1753-0407.70230)
Supplement: Supplementary file 1 — Appendix S1: Supporting Information [file JDB-18-e70230-s001.docx]

Mortality from cancer, from cardiovascular disease, and from dementia separately analyzed for people with diabetes and people without diabetes in 2000, 2005, 2010, 2015 and 2020

Graphs and Table from Magliano DJ, Morton JI, Chen L, Sacre JW, Salim A, Gong JY, Carstensen B, Gregg EW, Arffman M, Booth GL, Ter Braake JG, Chu LM, Eeg-Olofsson K, Eliasson B, Fleetwood K, Fosse-Edorh S, Garbuviene M, Guion M, Ha KH, Kaul P, Ke C, Keskimäki I, Kim DJ, Laurberg T, Støvring H, Vos RC, Wild SH, Shaw JE. Trends in cause-specific mortality among people with and without diabetes in high-income settings: a multinational, population-based study. Lancet Diabetes Endocrinol. 2026 Mar 9:S2213-8587(25)00398-5. doi: 10.1016/S2213-8587(25)00398-5. Supplementary appendix 1

First prompt:

Analyze the graphic data from Supplementary figures 2 (Cancer morality), 3 (CVD mortality), and 5 (Dementia mortality), using at the top panels for people with diabetes and people not having diabetes combining males and females. Combine this with the data of “Supplementary Table 6: Summary of data included in the analysis.” Create a table showing mortality from cancer per 1000 people with rows for each country and a summary row with mean from all the countries weighted by person years of followup for each country, with columns for mortality from cancer per 1000 people with diabetes and for mortality from cancer per 1000 people without diabetes separately for 2000, 2005, 2010, 2015 and 2020. Create a table showing mortality from cardiovascular disease per 1000 people with rows for each country and a summary row with mean from all the countries weighted by person years of followup for each country, with columns for mortality from cancer per 1000 people with diabetes and for mortality from cardiovascular disease per 1000 people without diabetes separately for 2000, 2005, 2010, 2015 and 2020. Create a table showing mortality from dementia per 1000 people with rows for each country and a summary row with mean from all the countries weighted by person years of followup for each country, with columns for mortality from dementia per 1000 people with diabetes and for mortality from cancer per 1000 people without diabetes separately for 2000, 2005, 2010, 2015 and 2020. For all tables, if data for one of the countries is not available for a given row and column, place a dash, and do not use that item in summary calculation.

**Mortality from Cancer, Cardiovascular Disease, and Dementia by Country, 2000–2020**

*Source: Magliano DJ et al. Lancet Diabetes Endocrinol. 2026. Supplementary appendix.*

**General Notes**

• All values are mortality rates per 1,000 person-years.

• "—" indicates the country's study period did not include that year.

• The "Weighted mean" row uses person-years of follow-up as weights; countries with "—" for a given cell are excluded from that cell's calculation.

• Netherlands and Sweden are not included (proportional mortality analysis only).

**Table 1. Cancer Mortality (per 1,000 person-years)**

| **Country** | **With Diabetes** | | | | | **Without Diabetes** | | | | |
| --- | --- | --- | --- | --- | --- | --- | --- | --- | --- | --- |
|  | **2000** | **2005** | **2010** | **2015** | **2020** | **2000** | **2005** | **2010** | **2015** | **2020** |
| Alberta, Canada | — | 9.0 | 8.2 | 7.5 | 6.5 | — | 6.0 | 5.5 | 5.2 | 5.0 |
| Australia | — | 9.5 | 8.5 | 7.5 | 7.0 | — | 5.5 | 5.0 | 5.0 | 5.2 |
| Denmark | — | 12.0 | 10.5 | 9.0 | — | — | 8.5 | 7.5 | 7.0 | — |
| Finland | 9.0 | 8.5 | 8.0 | 7.5 | 7.0 | 6.5 | 6.2 | 5.8 | 5.5 | 4.8 |
| France | — | — | — | 7.5 | 7.0 | — | — | — | 5.0 | 4.8 |
| Lithuania | — | — | — | 9.0 | 8.5 | — | — | — | 6.0 | 6.5 |
| Ontario, Canada | — | — | — | 6.0 | — | — | — | — | 4.5 | — |
| Scotland | — | — | 9.5 | 9.0 | 9.0 | — | — | 6.2 | 6.2 | 7.5 |
| South Korea | — | — | 6.5 | 5.5 | — | — | — | 2.5 | 2.0 | — |
| **Weighted mean** | **9.0** | **9.5** | **8.6** | **7.5** | **7.1** | **6.5** | **6.2** | **5.6** | **5.2** | **5.2** |

*Values are modeled mortality rates read from published graphs (Magliano et al., 2026). Weighted mean uses person-years of follow-up as weights; countries without data for a given year are excluded from that calculation.*

**Table 2. Cardiovascular Disease Mortality (per 1,000 person-years)**

| **Country** | **With Diabetes** | | | | | **Without Diabetes** | | | | |
| --- | --- | --- | --- | --- | --- | --- | --- | --- | --- | --- |
|  | **2000** | **2005** | **2010** | **2015** | **2020** | **2000** | **2005** | **2010** | **2015** | **2020** |
| Alberta, Canada | — | 15.0 | 11.0 | 9.0 | 7.5 | — | 6.5 | 5.0 | 4.0 | 3.5 |
| Australia | — | 11.0 | 9.0 | 7.5 | 6.0 | — | 6.0 | 5.0 | 4.0 | 3.5 |
| Denmark | — | 16.0 | 13.0 | 10.0 | — | — | 10.0 | 8.0 | 6.0 | — |
| Finland | 23.0 | 18.0 | 14.0 | 12.0 | 10.0 | 13.0 | 11.0 | 8.0 | 7.0 | 6.0 |
| France | — | — | — | 7.5 | 5.5 | — | — | — | 3.5 | 2.5 |
| Lithuania | — | — | — | 23.0 | 21.0 | — | — | — | 19.0 | 18.0 |
| Ontario, Canada | — | — | — | 7.5 | — | — | — | — | 3.5 | — |
| Scotland | — | — | 11.0 | 10.0 | 10.0 | — | — | 5.0 | 5.0 | 4.5 |
| South Korea | — | — | 6.0 | 5.0 | — | — | — | 3.5 | 2.5 | — |
| **Weighted mean** | **23.0** | **14.3** | **11.1** | **8.7** | **7.2** | **13.0** | **7.8** | **5.9** | **4.5** | **3.7** |

*Values are modeled mortality rates read from published graphs (Magliano et al., 2026). Weighted mean uses person-years of follow-up as weights; countries without data for a given year are excluded from that calculation.*

**Table 3. Dementia Mortality (per 1,000 person-years)**

| **Country** | **With Diabetes** | | | | | **Without Diabetes** | | | | |
| --- | --- | --- | --- | --- | --- | --- | --- | --- | --- | --- |
|  | **2000** | **2005** | **2010** | **2015** | **2020** | **2000** | **2005** | **2010** | **2015** | **2020** |
| Alberta, Canada | — | 1.3 | 1.5 | 1.6 | 1.7 | — | 0.7 | 1.0 | 1.2 | 1.3 |
| Australia | — | 0.8 | 1.2 | 1.5 | 1.6 | — | 1.0 | 1.1 | 1.2 | 1.3 |
| Denmark | — | 0.9 | 1.2 | 1.8 | — | — | 0.9 | 1.1 | 1.5 | — |
| Finland | 2.1 | 2.2 | 2.8 | 3.5 | 4.5 | 2.0 | 2.1 | 2.5 | 3.0 | 3.5 |
| France | — | — | — | 0.7 | 0.7 | — | — | — | 0.9 | 0.9 |
| Lithuania | — | — | — | 0.3 | 0.3 | — | — | — | 0.1 | 0.2 |
| Ontario, Canada | — | — | — | 1.3 | — | — | — | — | 1.0 | — |
| Scotland | — | — | 1.5 | 1.8 | 2.3 | — | — | 1.3 | 1.7 | 2.1 |
| South Korea | — | — | 0.5 | 0.5 | — | — | — | 0.1 | 0.1 | — |
| **Weighted mean** | **2.1** | **1.3** | **1.7** | **1.5** | **1.7** | **2.0** | **1.2** | **1.3** | **1.3** | **1.4** |

*Values are modeled mortality rates read from published graphs (Magliano et al., 2026). Weighted mean uses person-years of follow-up as weights; countries without data for a given year are excluded from that calculation.*

Second prompt:

Now create a table with cancer mortality rate ratio of people with diabetes to that of people without diabetes separately for 2000, 2005, 2010, 2015 and 2020. Create a table with cardiovascular disease mortality rate ratio of people with diabetes to that of people without diabetes separately for 2000, 2005, 2010, 2015 and 2020. Create a table with dementia mortality rate ratio of people with diabetes to that of people without diabetes separately for 2000, 2005, 2010, 2015 and 2020.

**Mortality Rate Ratios (Diabetes vs. No Diabetes) for Cancer, Cardiovascular Disease, and Dementia by Country, 2000–2020**

*Source: Magliano DJ et al. Lancet Diabetes Endocrinol. 2026. Supplementary appendix.*

**General Notes**

- Rate ratio = mortality rate in people with diabetes ÷ mortality rate in people without diabetes.
- Values are derived from modeled mortality rates read from published log-scale graphs.
- “—” indicates the country’s study period did not include that year, or data were not available.
- Weighted mean uses person-years of follow-up (diabetes group) as weights; countries with “—” for a given year are excluded from that year’s calculation.
- Netherlands and Sweden are not included (proportional mortality analysis only).

**Table 1. Cancer Mortality Rate Ratio (Diabetes / No Diabetes)**

| **Country** | **2000** | **2005** | **2010** | **2015** | **2020** |
| --- | --- | --- | --- | --- | --- |
| Alberta, Canada | — | 1.50 | 1.49 | 1.44 | 1.30 |
| Australia | — | 1.73 | 1.70 | 1.50 | 1.35 |
| Denmark | — | 1.41 | 1.40 | 1.29 | — |
| Finland | 1.38 | 1.37 | 1.38 | 1.36 | 1.46 |
| France | — | — | — | 1.50 | 1.46 |
| Lithuania | — | — | — | 1.50 | 1.31 |
| Ontario, Canada | — | — | — | 1.33 | — |
| Scotland | — | — | 1.53 | 1.45 | 1.20 |
| South Korea | — | — | 2.60 | 2.75 | — |
| **Weighted mean** | **1.38** | **1.55** | **1.57** | **1.47** | **1.40** |

*Values are modeled mortality rates read from published graphs (Magliano et al., 2026). Rate ratio = mortality in people with diabetes / mortality in people without diabetes. Weighted mean uses person-years of follow-up as weights.*

**Table 2. Cardiovascular Disease Mortality Rate Ratio (Diabetes / No Diabetes)**

| **Country** | **2000** | **2005** | **2010** | **2015** | **2020** |
| --- | --- | --- | --- | --- | --- |
| Alberta, Canada | — | 2.31 | 2.20 | 2.25 | 2.14 |
| Australia | — | 1.83 | 1.80 | 1.88 | 1.71 |
| Denmark | — | 1.60 | 1.62 | 1.67 | — |
| Finland | 1.77 | 1.64 | 1.75 | 1.71 | 1.67 |
| France | — | — | — | 2.14 | 2.20 |
| Lithuania | — | — | — | 1.21 | 1.17 |
| Ontario, Canada | — | — | — | 2.14 | — |
| Scotland | — | — | 2.20 | 2.00 | 2.22 |
| South Korea | — | — | 1.71 | 2.00 | — |
| **Weighted mean** | **1.77** | **1.80** | **1.85** | **1.98** | **1.97** |

*Values are modeled mortality rates read from published graphs (Magliano et al., 2026). Rate ratio = mortality in people with diabetes / mortality in people without diabetes. Weighted mean uses person-years of follow-up as weights.*

**Table 3. Dementia Mortality Rate Ratio (Diabetes / No Diabetes)**

| **Country** | **2000** | **2005** | **2010** | **2015** | **2020** |
| --- | --- | --- | --- | --- | --- |
| Alberta, Canada | — | 1.86 | 1.50 | 1.33 | 1.31 |
| Australia | — | 0.80 | 1.09 | 1.25 | 1.23 |
| Denmark | — | 1.00 | 1.09 | 1.20 | — |
| Finland | 1.05 | 1.05 | 1.12 | 1.17 | 1.29 |
| France | — | — | — | 0.78 | 0.78 |
| Lithuania | — | — | — | 3.00 | 1.50 |
| Ontario, Canada | — | — | — | 1.30 | — |
| Scotland | — | — | 1.15 | 1.06 | 1.10 |
| South Korea | — | — | 5.00 | 5.00 | — |
| **Weighted mean** | **1.05** | **1.04** | **1.28** | **1.16** | **1.05** |

*Values are modeled mortality rates read from published graphs (Magliano et al., 2026). Rate ratio = mortality in people with diabetes / mortality in people without diabetes. Weighted mean uses person-years of follow-up as weights.*

Third prompt:

Now create a table with rate of cardiovascular disease mortality to that of cancer mortality among people with diabetes, and rate of cardiovascular disease mortality to that of cancer mortality among people without diabetes separately for 2000, 2005, 2010, 2015 and 2020. Create a table with rate of cardiovascular disease mortality to that of dementia mortality among people with diabetes, and rate of cardiovascular disease mortality to that of dementia mortality among people without diabetes separately for 2000, 2005, 2010, 2015 and 2020. Create a table with rate of cancer mortality to that of dementia mortality among people with diabetes, and rate of cancer mortality to that of dementia mortality among people without diabetes separately for 2000, 2005, 2010, 2015 and 2020.

**Cause-Specific Mortality Rate Ratios by Country, 2000–2020**

*Source: Magliano DJ et al. Lancet Diabetes Endocrinol. 2026. Supplementary appendix.*

**General Notes**

- Each table shows the ratio of mortality rates between two causes of death, computed separately for people with diabetes and people without diabetes.
- Values are derived from modeled mortality rates read from published log-scale graphs.
- "—" indicates the country's study period did not include that year, or data were not available.
- Weighted mean uses person-years of follow-up as weights (diabetes person-years for the "With Diabetes" column; non-diabetes person-years for the "Without Diabetes" column); countries with "—" for a given year are excluded from that year's calculation.
- Netherlands and Sweden are not included (proportional mortality analysis only).

**Table 1. CVD / Cancer Mortality Rate Ratio**

| **Country** | **With Diabetes** | | | | | **Without Diabetes** | | | | |
| --- | --- | --- | --- | --- | --- | --- | --- | --- | --- | --- |
|  | **2000** | **2005** | **2010** | **2015** | **2020** | **2000** | **2005** | **2010** | **2015** | **2020** |
| Alberta, Canada | — | 1.67 | 1.34 | 1.20 | 1.15 | — | 1.08 | 0.91 | 0.77 | 0.70 |
| Australia | — | 1.16 | 1.06 | 1.00 | 0.86 | — | 1.09 | 1.00 | 0.80 | 0.67 |
| Denmark | — | 1.33 | 1.24 | 1.11 | — | — | 1.18 | 1.07 | 0.86 | — |
| Finland | 2.56 | 2.12 | 1.75 | 1.60 | 1.43 | 2.00 | 1.77 | 1.38 | 1.27 | 1.25 |
| France | — | — | — | 1.00 | 0.79 | — | — | — | 0.70 | 0.52 |
| Lithuania | — | — | — | 2.56 | 2.47 | — | — | — | 3.17 | 2.77 |
| Ontario, Canada | — | — | — | 1.25 | — | — | — | — | 0.78 | — |
| Scotland | — | — | 1.16 | 1.11 | 1.11 | — | — | 0.81 | 0.81 | 0.60 |
| South Korea | — | — | 0.92 | 0.91 | — | — | — | 1.40 | 1.25 | — |
| **Weighted mean** | **2.56** | **1.54** | **1.30** | **1.16** | **1.00** | **2.00** | **1.24** | **1.06** | **0.86** | **0.71** |

*CVD mortality rate divided by cancer mortality rate, computed separately for people with and without diabetes. Values derived from modeled rates (Magliano et al., 2026). Weighted mean uses person-years of follow-up as weights.*

**Table 2. CVD / Dementia Mortality Rate Ratio**

| **Country** | **With Diabetes** | | | | | **Without Diabetes** | | | | |
| --- | --- | --- | --- | --- | --- | --- | --- | --- | --- | --- |
|  | **2000** | **2005** | **2010** | **2015** | **2020** | **2000** | **2005** | **2010** | **2015** | **2020** |
| Alberta, Canada | — | 11.54 | 7.33 | 5.62 | 4.41 | — | 9.29 | 5.00 | 3.33 | 2.69 |
| Australia | — | 13.75 | 7.50 | 5.00 | 3.75 | — | 6.00 | 4.55 | 3.33 | 2.69 |
| Denmark | — | 17.78 | 10.83 | 5.56 | — | — | 11.11 | 7.27 | 4.00 | — |
| Finland | 10.95 | 8.18 | 5.00 | 3.43 | 2.22 | 6.50 | 5.24 | 3.20 | 2.33 | 1.71 |
| France | — | — | — | 10.71 | 7.86 | — | — | — | 3.89 | 2.78 |
| Lithuania | — | — | — | 76.67 | 70.00 | — | — | — | 190.00 | 90.00 |
| Ontario, Canada | — | — | — | 5.77 | — | — | — | — | 3.50 | — |
| Scotland | — | — | 7.33 | 5.56 | 4.35 | — | — | 3.85 | 2.94 | 2.14 |
| South Korea | — | — | 12.00 | 10.00 | — | — | — | 35.00 | 25.00 | — |
| **Weighted mean** | **10.95** | **12.29** | **7.34** | **8.26** | **6.67** | **6.50** | **7.05** | **5.23** | **6.97** | **4.38** |

*CVD mortality rate divided by dementia mortality rate, computed separately for people with and without diabetes. Values derived from modeled rates (Magliano et al., 2026). Weighted mean uses person-years of follow-up as weights. Lithuania and South Korea have very low dementia mortality rates, producing large ratios.*

**Table 3. Cancer / Dementia Mortality Rate Ratio**

| **Country** | **With Diabetes** | | | | | **Without Diabetes** | | | | |
| --- | --- | --- | --- | --- | --- | --- | --- | --- | --- | --- |
|  | **2000** | **2005** | **2010** | **2015** | **2020** | **2000** | **2005** | **2010** | **2015** | **2020** |
| Alberta, Canada | — | 6.92 | 5.47 | 4.69 | 3.82 | — | 8.57 | 5.50 | 4.33 | 3.85 |
| Australia | — | 11.88 | 7.08 | 5.00 | 4.38 | — | 5.50 | 4.55 | 4.17 | 4.00 |
| Denmark | — | 13.33 | 8.75 | 5.00 | — | — | 9.44 | 6.82 | 4.67 | — |
| Finland | 4.29 | 3.86 | 2.86 | 2.14 | 1.56 | 3.25 | 2.95 | 2.32 | 1.83 | 1.37 |
| France | — | — | — | 10.71 | 10.00 | — | — | — | 5.56 | 5.33 |
| Lithuania | — | — | — | 30.00 | 28.33 | — | — | — | 60.00 | 32.50 |
| Ontario, Canada | — | — | — | 4.62 | — | — | — | — | 4.50 | — |
| Scotland | — | — | 6.33 | 5.00 | 3.91 | — | — | 4.77 | 3.65 | 3.57 |
| South Korea | — | — | 13.00 | 11.00 | — | — | — | 25.00 | 20.00 | — |
| **Weighted mean** | **4.29** | **8.98** | **6.11** | **7.10** | **6.72** | **3.25** | **5.96** | **4.97** | **5.64** | **4.84** |

*Cancer mortality rate divided by dementia mortality rate, computed separately for people with and without diabetes. Values derived from modeled rates (Magliano et al., 2026). Weighted mean uses person-years of follow-up as weights. Lithuania and South Korea have very low dementia mortality rates, producing large ratios.*
